# Histopathological Changes by the Use of Soft Reline Materials: A Rat Model Study

**DOI:** 10.1371/journal.pone.0100293

**Published:** 2014-06-25

**Authors:** Michele Bail, Lissandra Matos Brol Meister, Eduardo Bauml Campagnoli, Janaina Habib Jorge, Manuella de Cassia Iglesias Ban, Alfonso Sanchez-Ayala, Nara Hellen Campanha

**Affiliations:** 1 PhD Student, Department of Dentistry, State University of Ponta Grossa, Ponta Grossa, Parana, Brazil; 2 Professor, Department of Dentistry, State University of Ponta Grossa, Ponta Grossa, Parana, Brazil; 3 Professor, Department of Dental Materials and Prosthodontics, Araraquara Dental School, Univ. Estadual Paulista, UNESP, Araraquara, São Paulo, Brazil; 4 Undergraduate Student, Department of Dentistry, State University of Ponta Grossa, Ponta Grossa, Parana, Brazil; University of North Carolina at Chapel Hill, United States of America

## Abstract

**Aim:**

To assess the histopathological changes of rat palatal mucosa exposed to soft reline materials.

**Methods:**

Forty-five adult female Wistar rats with controlled living conditions and fed *ad libitum* were employed. Palatal appliances of heat-polymerized acrylic resin Lucitone 550 were manufactured and worn by forty animals during 14 days. Five animals did not use the appliances (G1) and were used to control the appliance influence. One experimental group (n = 10) used the appliances without any relining material (G2) to control the material effect. Three experimental groups (n = 10) received the following soft reline materials below appliances: Dentusoft (G3), Dentuflex (G4), and Trusoft (G5). Appliances from half of each experimental group(n = 5) was immersed in water bath at 55°C for 10 min before use. Animals were slaughtered and the palates were fixed in 10% buffered formalin. Hematoxylin and eosin stained sections of 5 µm were analyzed by computerized planimetry. Cellular compartment, keratin and total epithelial thickness, and basement membrane length were measured to histopathological description.Analysis of variance and Tukey post-hoc test were used to data examination(α = 0.05).

**Results:**

For heat-treatment groups, G4 showed less elongated ridges at the basal layer interface (*p* = 0.037) than G2. When comparing the conditions with and without heat-treatment, only G2 showed a significant decrease in the cellular compartment, keratin layer and total epithelium thickness (*p*<0.05).

**Conclusion:**

The post-polymerization for Lucitone 550 was an effective method to reduce the changes in the rat palatal mucosa. The soft reline materials tested did not cause significant histopathological changes in the rat palatal mucosa.

## Introduction

Resilient liners are recommended to decrease traumas caused by the complete denture on the oral mucosa, to fibromucosous tissue conditioning, denture stabilizing and ridge healing after surgery, or as a temporary base material during the osseointegration of dental implants, and several procedures to implant-retained overdentures holding [1]–[6]. Relining materials may be classified based on time of use as temporary and permanent. They may also be divided according to their composition in acrylic-based or silicone-based materials [1], [6].

The acrylic-based resilient lining materials recommended for relining technique are mostly auto-polymerized materials comprising a liquid mixture of an aromatic ester and ethanol interfused with high polymers [7]. The addition of phthalates is aimed at increasing flexibility, extensibility and to enhance working properties [6]–[9]. Although low or no toxicity related to these compounds has been reported, they possess phthalates, potentially toxic compounds which may promote undesirable biological effects [10] such as reducing male fertility [11], functioning as xenoestrogens [12]–[13] inducing hormonal tumors, and causing fetal malformations [14]–[15].

There is higher residual monomer in auto-polymerizing acrylic-based materials [16]–[19] caused by the low degree of conversion achieved by the use of chemical activator [17]. Free radical polymerization reaction does not result in complete conversion of all carbon–carbon double bonds and therefore, acrylic resins are known to contain and release unpolymerized monomers [7], [20]. The leachability of plasticizers (such as phthalates) released in water [21]–[22] and residual monomer may also induce a direct cytotoxic effect on cells [23]–[26]. Toxic compounds may be released into the patient's mouth during the polymerization of soft reline materials in contact with the oral mucosa in the relining procedure. Consequently, reactions of the oral mucosa to acrylic materials, such as “burning mouth sensation” [27], redness, swelling and pain in the oral mucosa [28], have been described. Moreover, a clinical case was reported as being the first case of isolated systemic allergic reaction caused by acrylic-based materials [29].Possibly these reactions were produced by the release of unpolymerized monomers [7], [20].

Post-polymerization heat treatment has been shown to be an effective method in reducing the amount of residual monomers emanating from acrylic resins [19], [30]. This reduction of residual monomers could, in theory, decrease the release of toxic residual denture reline materials, and consequently decrease the potential to cause irritation, allergic response and symptoms due to the previous elimination of these substances. Studies in the literature demonstrated a reduction in the cytotoxicity of acrylic resin for denture base when a post-polymerization heat treatment at 55°C for 60 min was applied [31], and also the cytotoxicity of a hard lining material decreased when applying a post-polymerization heat treatment at 55°C for 10 minutes [24].

There are few current in vivo studies that have evaluated soft reline materials with different compositions and their effects on the epithelium [32]. It has been observed that relining with either a soft or a hard relining material gave an increased thickness of keratin, and it was supposed that relining may have had an effect on the rate of transition of cells through the epithelium, with a more rapid formation of keratin [32]. Barclay et al. [32] encountered problems in fixing palatal appliances to the incisor teeth with relining materials and they reported a failure rate of 65% percent of the appliances, mostly within 24 h of their fixing stage. Based on the literature report, our group used a new rat model to fix acrylic resin plates (in press). The aim of this work was to assess histopathological changes using soft reline materials, with and without post–polymerization heat treatment.

## Material and Methods

### Experimental design

Experimental groups and materials used are presented in Table 1. The composition and batch number of the materials are also summarized in the same table. Forty five adult female rats (*Rattus norvegicus albinus*, wistar), aged sixty days, and weighing approximately 200 g were used in this study. The animals were maintained in separate cages at 23°C with 56% relative humidity, alternating light/dark cycles of 12 h. The animals were fed *ad libitum* with water and a nutritionally complete powdered feed (Nuvilab CR-1, Nuvital, PR, Curitiba, Brazil). This study was approved in accordance with the recommendations of the Ethics Committee on Animal Research of Ponta Grossa State University (Protocol 12673/2010). The guidelines on animal handling of the COBEA (Brazilian College of Animal Experimentation) were also followed.

**Table 1:** Materials selected for the study.

Material	Code	Manufacter	Powder/liquid ratio	Composition	Type and curing	Batch Number
Lucitone 550	L	Dentisply-Indústria e comércio Ltda, Petrópolis, Brazil	2.1 g/1 mL	Powder – PMMA; Liquid – MMA and EDGMA	Termopolymerized hard acrylic resin	266775C
Dentusoft (Tissue conditioner)	DS	Densell, Buenos Aires, Argentina	2 g/1 mL	Powder: Polietil methacrylate, benzoylperoxide, titanium oxide; Liquid: Dibutyl phthalate, alcohol.	Auto-polimerized plasticized acrylic	IB0083
Dentuflex	DF	Densell, Buenos Aires, Argentina	2 g/1 mL	Powder: Polietil methacrylate, benzoyl peroxide; Liquid: N-Butyl methacrylate, Dibutyl phthalate, Trimethylolpropane, Dimethyl-p-toluidine	Auto-polimerized plasticized acrylic	HL0645
Trusoft	TS	The Bosworth Co., Skokie, USA	1.06 g/1 mL	Powder: Polietil methacrylate, cadmium pigments; Liquid: Ethyl alcohol, dibutyl phthalate	Auto-polimerized plasticized acrylic	1007–333

### Acrylic plate preparation

One week prior to the impression stage, the animals started to receive a paste diet obtained from mixing the powdered diet with enough water to form a thick paste. They had free access to water for 12 h before the experimental procedures. They were anesthetized by the administration of a solution of ketamin (100 mg/mL) and xylasine (100 mg/mL) that was made by diluting 3.75 mL of ketamin plus 0.5 mL of xylasine in 5.75 mL of distilled water. Intraperitonial injection of 0.2 mL/100 g of this solution was used to sedate the rats, providing the working time of approximately 30 min for procedures. A special support kept the animals' mouths open while they were anesthetized, allowing an appropriate position for the handling of the animals.

After sedation, each rat was weighed (w^i^) using an analytical balance (Gehaka, Ind. e Com. Eletro, Eletrônica Gehaka Ltda, São Paulo, Brazil). After obtaining the master impression by using an addition silicone material (Futura, DFL, RJ, Rio de Janeiro, Brazil), a pattern of plates was waxed, covering the palate and the molars of the stone casts (thick 1 mm). The casts with wax patterns in position were conventionally included and the denture base acrylic resin Lucitone 550 (Dentsply, Petrópolis, RJ, Brazil) was heat-polymerized according to the manufacturer's protocol. After polymerization, the flasks were bench cooled at room temperature for 30 min and 15 min under running water before the specimens were removed. The palatal plates were visually inspected to verify that they presented a smooth surface and showed no voids or porosity; otherwise, they were discarded. The edges of the denture base were also carefully finished to remove irregularities. The plates of acrylic resin were stored in 50 mL of distilled water for 48 h at 37°C prior to insertion, in order to eliminate the possible effects of any residual monomer [33]–[34].

### Palatal plate relining and fixing

**Figure 1:**
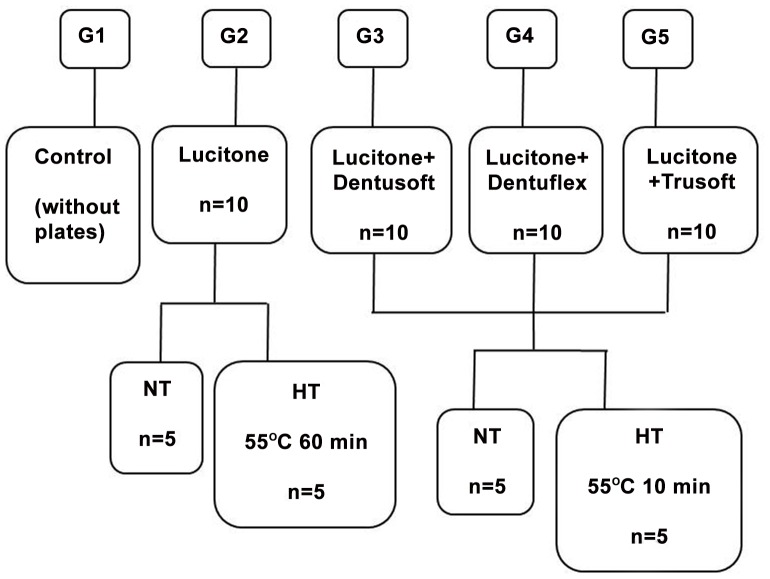
Diagram representation of the experimental groups. NT: no treatment; HT: with heat-treatment.

The acrylic plates of the groups G2, G3, G4 and G5 were relined with the materials according to Figure 1.

The acrylic custom-made palatal plates (Lucitone 550) were relined with the resilient liners of each group from dynamic impressions (n = 10). Half of the animals of each experimental group (n = 5) were installed without post-polymerization treatment (NT), and the other groups were immersed in water-bath (HT) at 55°C for 10 minutes (G3, G4, G5; n = 5) or for 60 minutes (G2; n = 5) before the fixing stage. All soft materials were prepared in accordance with the manufacturers' recommendations, and the plates were placed on the palate with the respective material mixture until its complete polymerization. After polymerization, excess material beyond the borders of the plate was carefully removed. In order to promote a mechanical retention and fixing of the palatal plate, a groove in the molar coverage region was made using a spherical bur #6. Composite filling material (Opallis, FGM, Joinville, SC, Brazil) was used to fix the devices [34] by filling the molar region groove. The composite provided only a mechanical retention to the molars, since no adhesive system was applied. The animals used the palatal plates for 14 days and were kept on a paste diet. The period was determined according to ISO 10993 [35] and ISO 7405 [36]. In addition, this period is recommended as the maximum time a soft reline material should be in contact with the mucosa.

### Histology and morphological quantitative analysis

After 14 days, the animals were weighed (w^f^) and killed according to the standards of the Brazilian Society of Laboratory Animal Science (SBCAL-COBEA). Acrylic palatal-plates were removed, and the palates were dissected from the heads and fixed in 10% buffered formalin for a minimum of 24 h, and a maximum of 48 h. The blocks were demineralized in 4.13% EDTA and then were cut in the sagittal plane posterior to the molars. The blocks were processed and paraffin embedded for sections to be cut from the posterior side of each block. Sections of 5 µm (Leica, Berlin, Germany) were stained with haematoxylin and eosin (HE). Slides were mounted and observed by light microscopy (Nikon – model YS 100, Tokyo - Japan). Two standardized photos (200 x) of two histological fields were selected: the field of the epithelium overlying the palatal blood vessels (L), and the field at the midline palatine raphe (M). Tissue reactions such as inflammatory infiltrate, fibroblast proliferation, and vascular changes were assessed in the stained sections (HE). Changes in epithelial tissue morphology were assessed by computerized planimetry using the software Image Pro Plus, Version 4.5.0.29 (Media Cybernetics, Inc, Bethesda, USA). Within each field, the length of the basal layer interface (BL) was measured in micrometers. In addition, keratinized compartment (KC) and cellular compartment (CC) were measured using a standardized grid of 10 horizontal lines, which gave a mean of 10 measurements of each photo. The addition of the keratinized compartment value (KC) to the cellular compartment value (CC) resulted in the total epithelium mean (TE).

### Statistical analysis

All data were analyzed using IBM® SPSS® Statistics 20 software. The weight of the animals (both initially and prior to killing) was contrasted using Wilcoxon signed-rank sum test. As regards to histology and morphological quantitative analysis, measurements of the two analyzed regions were averaged to each variable as a method of improving the consistency of the means. Normality of residuals and homogeneity of variances were examined by Shapiro-Wilk and Levene's tests, respectively. One-way analysis of variance and Tukey's post-hoc tests were used to compare the biocompatibility of resilient lining materials according to cellular compartment, keratin thickness, epithelial surface length and epithelium morphology values, and basement membrane length results after base-10 logarithmic transformation. This procedure was also applied to the analysis of resilient lining materials after post-polymerization treatment regarding cellular compartment, basement membrane length, and total epithelial thickness data. Variables presenting heteroscedasticity and non-normal distribution were studied employing one-way analysis of variance and non-parametric multiple comparisons tests. Student's t-test was used to compare values from resilient lining materials with and without post-polymerization treatment for all variables. All statistical inferences were performed with two-tailed trials assuming a 5% significance level.

## Results

During the experiment four animals, two of them from the G4 group and one from the G4 and G3 groups respectively, had premature plate loss. Moreover, there were three anesthetic-related deaths among all female adult rats used in this study. These animals were immediately substituted in order to keep the number of samples (n) in each group. Weights were recorded throughout the experiment, one week after the animals commenced the paste diet (w^i^) and 14 days after the fitting of appliances (w^f^). The mean weights are demonstrated in Table 2.The w^i^ (g) ranged from 182.20 to 211.00 and the w^f^ (g) were from 181.20 to 230.00. The mean weights were found to rise in the G1 group (*p* = 0.043) and in the G5 group (*p* = 0.043). For the G2, G3 and G4 groups there were no statistical differences for the mean weights w^i^ and w^f^ (*p*≥0.05).

**Table 2:** Mean ± standard deviation (g) of animal weights (n = 5).

Resilient lining material	Initially (wi)	Previously to sacrifice (wf)	P
Control	211.00±15.166b	230.60±8.62a	0.043
Lucitone	220.00±12.25b	209.20±17.21b	0.138
Dentusoft	182.20±14.72b	181.20±40.29b	0.686
Dentuflex	193.20±12.48b	184.80±23.64b	0.500
Trusoft	194.00±6.44b	220.00±10.93a	0.043

Wilcoxon test: Horizontally, means with identical letters were not significantly different (*P*≥0.05).

**Figure 2:**
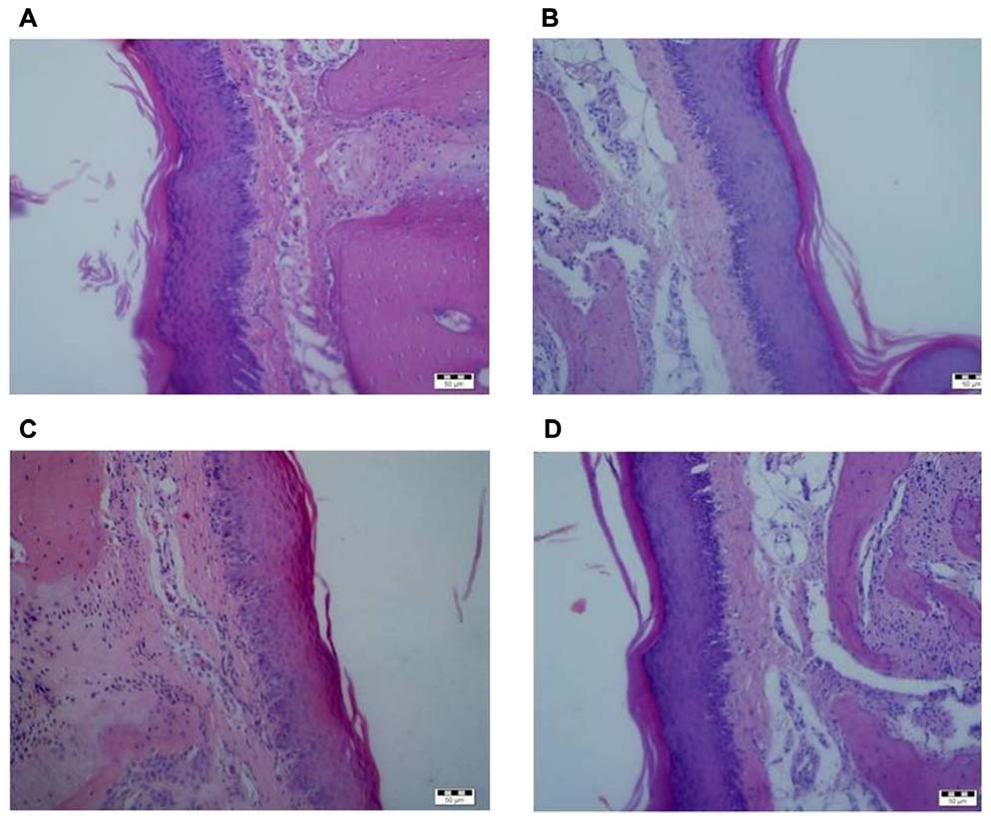
Photomicrograph of paraffin sections of Wistar rat keratinized palatal oral mucosa after hematoxylin eosin staining (200X) (A = group G2, B =  group G3, C =  group G4, D =  group G5).

**Figure 3:**
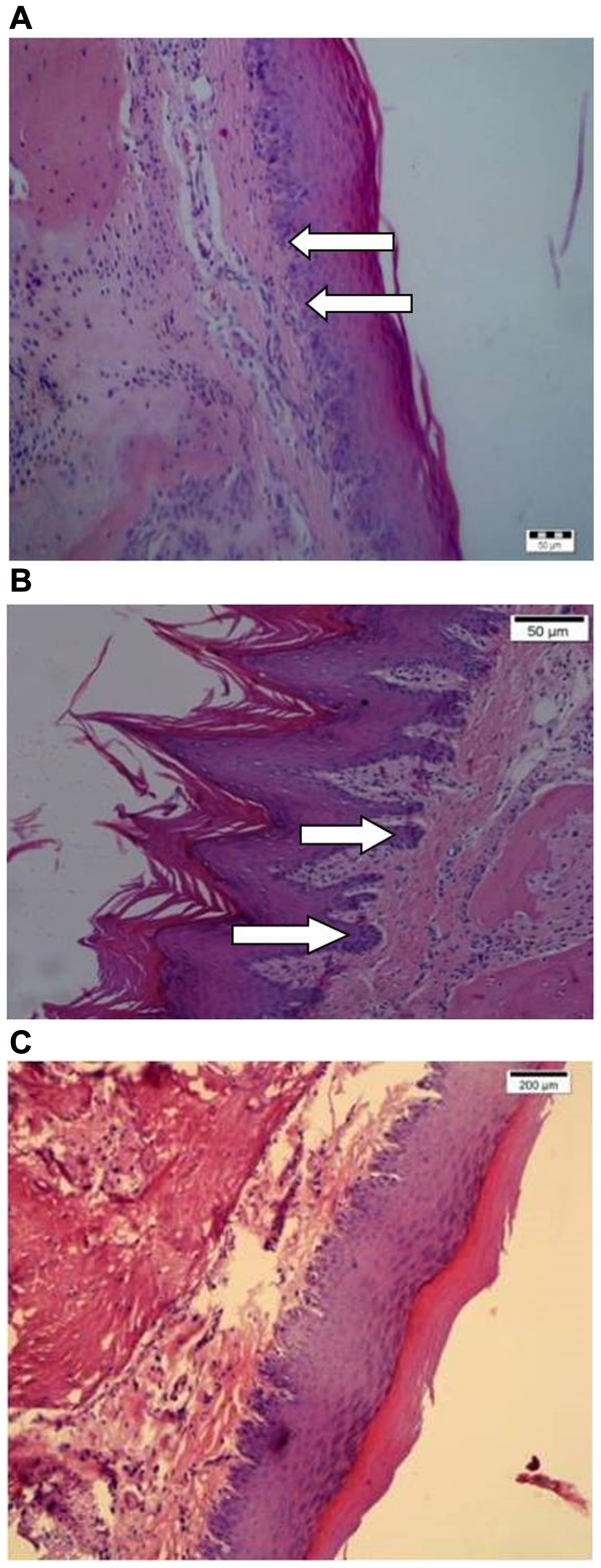
Photomicrograph of paraffin sections of Wistar rat keratinized palatal oral mucosa after hematoxylin eosin staining (200X)(A =  Dentuflex HT, B =  Lucitone 550 HT). In a arrowheads indicate the basal layer interface ridges, and in B the elongated basal layer interface ridges. In C: Control group (without plates).

Tissue reactions were qualitatively scored, such as inflammatory response, fibroblast proliferation, or vascular changes were not observed, as demonstrated in Figures 2 (A-B-C-D) and 3 (A-B) when compared to the control group (Figure 3-C).

The results from NT groups are shown in Table 3. Assessment of the thickness of keratin (KC - µm) ranged from 10.46 (±11.06) to 69.29 (±27.84) although statistical differences were not found among the materials tested, nor when compared with the control group (*p* = 0.075). The BL was not statistically different (*p*≥0.05). There were no significant differences in total epithelial thickness (TE) between materials tested or between test groups and control group (*p*≥0.05).

**Table 3:** Mean ± standard deviation (µm) of tissue parameters from resilient lining materials with no post polymerization treatment (n = 5).

Tissue parameters	Control	Lucitone	Dentusoft	Dentuflex	Trusoft	*P*
Cellular compartment	179.23±33.29	238.31±52.69	199.01±74.93	173.80±34.22	228.67±102.82	0.440
Keratinthickness	43.33±10.52	69.29±27.84	27.61±38.17	10.46±11.06	44.66±48.10	0.075
Total epithelial thickness	222.57±36.26	307.60±66.89	226.63±95.74	184.30±32.92	273.35±118.22	0.148
Basal layer interface length	2446.86±609.23	3089.41±1285.56	2891.64±772.49	2568.74±616.67	2969.20±801.06	0.721

ANOVA: The difference is not significant (*P*>0.05).

The results with post-polymerization heat treatment (HT) are shown in Table 4. Assessment of the thickness of keratin (KC - µm) ranged from 16.38 (±11.10) to 49.76 (±10.6) although statistical differences were not found among the materials tested, nor when compared with the control group (*p* = 0.068). Figure 3 illustrates the basal layer interface length (BM) that was statistically different when comparing the groups G2 and G4 (*p* = 0.037). There were no significant differences in total epithelial thickness (TE) between materials tested, or between test groups and control group (*p*≥0.05).

**Table 4:** Mean ± standard deviation (µm) of tissue parameters from resilient lining materials with post polymerization treatment (n = 5).

Tissue parameters	Control	Lucitone	Dentusoft	Dentuflex	Trusoft	*P*
Cellular compartment	179.23±33.29	130.90±27.67	233.86±92.08	211.50±25.91	165.43±88.81	0.115
Keratin thickness	43.33±10.52	36.02±10.63	49.76±10.67	16.38±11.10	38.61±41.94	0.068
Total epithelial thickness	222.57±36.26	166.92±35.75	283.62±86.96	227.87±34.69	204.04±120.07	0.180
Basal layer interface length	2446.86±609.23^AB^	2883.46±588.78^A^	2097.37±372.88^AB^	1878.77±385.20^B^	2470.37±421.53^AB^	***0.037***

ANOVA: Means followed by different letters are statistically different (*P<0.05*).

*P*-values from comparisons between NT and HT for all materials tested are shown in Table 5. The values of the thickness of keratin (KC - µm), total epithelium (TE), and cellular compartment were statistically higher for NT than HT only in the case of G2 (*p*<0.05).

**Table 5:** *P*-value from comparison between with and without the heat post polymerization for all materials tested (n = 5).

Tissue parameters	Lucitone	Dentusoft	Dentuflex	Trusoft
Cellularcompartment	0.004*	0.530	0.085	0.328
Keratinthickness	0.037*	0.247	0.423	0.838
Total epithelialthickness	0.003*	0.353	0.076	0.385
Basal layer interface length	0.753	0.072	0.067	0.253

Student's t-test: *Statistically significant difference.

## Discussion

Soft liners for relining prostheses are considered to be acrylic resins which contain additives [1], [6] that have been incompletely studied. Although there have been no observations of clinical problems attributable to the use of those compounds, there is experimental evidence of subtle toxicity [10], [26]. The cytotoxicity test using the method of cell culture is considered to be a preliminary test to evaluate the biocompatibility of a material. Notwithstanding their scientific importance, cytotoxicity tests are considered to be initial tests and it is difficult to extrapolate data obtained from patients [37]. However, the elicited host response in *in vivo* models remains crucial for the biocompatibility evaluation of medical devices [38]. Usage tests may be the next step to evaluate the biological behavior of materials. For this purpose, animal experimentation tests are still the only generally acceptable form of testing the biocompatibility of dental materials [39]. *In vivo* studies using animal models have the advantage of providing an assessment of the biocompatibility of dental materials prior to their use in humans. It is widely known that results obtained from animal studies should be extrapolated to humans with caution. Such caution is particularly justified in situations where metabolic interactions and immune responses are part of the process of formation of potentially harmful agents, such as carcinogens. With dental materials, metabolic interactions between the host and the material probably play only a minor role in determining their biocompatibility [39]–[41].

The oral mucosa is one the most important physiological barriers against environmental stresses, chemical damages, mechanical agents, and bacterial infections. In addition, the oral mucosa is a cornified stratified squamous epithelium, supported and fed by the underlying connective lamina propria. The continuous renewal process of maturation and differentiation of keratinocytes migrating from the deepest proliferative layer (basal layer) to the upper compartments, finally reaching the horny layer, is a process that guarantees the mechanical and structural integrity of the epithelium [42]. The oral mucosa under dentures usually present some changes in the continuous renewal process of maturation and differentiation of keratinocytes, and this process is observed varying as histological changes to clinical abnormalities [42]–[45] as a process of adaptation of the epithelium in order to protect the adjacent structures [46].

The aim of this study was to quantitatively assess the morphological parameters of keratin thickness (KC), total epithelial thickness (ET) and basement membrane interface length [28], to evaluate the epithelium response to the resilient liners. Since the increase of the thickness of the oral mucosa under the denture base is also considered a physiological response to mechanical loading [47]–[52], and no compressive forces was reproduced in this study because the plates were fixed in the molars, we may not expect any increased proliferative activity of the epithelial cells due the mechanical compression factors [49]–[52]. The histopathological changes assessed may be a response of the epithelium to the release of toxic substances [20]–[21], [26], [41], [52]–[54] and/or residual monomers [24], [31]. Despite the cytotoxicity reported *in vitro* [26], in this present *in vivo* study no histopathological changes of the epithelium were observed forlining resins tested without the application of post-polymerization heat treatment. In addition, no tissue reactions such as inflammatory infiltrate, fibroblast proliferation, and vascular changes were observed for all materials tested. The toxic compounds in contact with tissue during polymerization were probably confined to the superficial horny layer of the keratinized epithelium [55]. These results can be explained by the greater resistance of the oral mucosa to the toxic substances released, compared to the cell culture, while these compounds arein direct contact with the cells in the cytotoxicity tests [56].The composition of the lining materials, especially the type of monomer added, could also explain the absence of histopathological changes in the rats' palate.According to the manufacturer, the DS, DF and TS relines contain polietil methacrylate, n-butyl methacrylate and polietil methacrylaterespectively. In a study about the associations of monomer structures and cytotoxicity,it was concludedthat methacrylates seem to be more cytotoxicity than dimethacrylates monomers [57].

The denture base material Lucitone 550 with no post-polymerization heat treatment showed increased thickness of keratin, cellular compartment, and total epithelial thickness when comparing these tissue parameters with the group with the application of the post-polymerization heat treatment. Since the heat treatment with hot water after polymerization procedure favorable to reduce the amount of residual monomers and residual substances [19], [30], [31], [34], [58], the post-polymerization treatment has improved the biological properties of the denture base material Lucitone 550, and the proliferative response of the epithelium was not observed in order to protect the underlying tissues. However, the results showed that heat treatment did cause not histopathological changes by the use of soft reline materials. The differences can be attributed to the differences in chemical composition of conventional resins and soft acrylic resins [59], because of its indications and the directcontact with the tissues, this material should benon-irritating and non-toxic [60].

This *in vivo* study of biocompatibility was accomplished with a method developed by our research group (in press). In this study the experimental palatal plate of heat-polymerized acrylic resin Lucitone 550, fitting the animal's palate and covering all molars, showed a failure rate of 10% percent of the plates, observed at the moment of the animal's death. The appliances reported by Barclay et al. [32] were relined with a resilient soft lining material and a hard chairside reline material. The devices were fixed in the palate of the rats by a retentive element encompassing the upper incisor teeth that were constructed from a band of 4 mm-wide stainless steel. The wire loop acted as retention for the acrylic. In the aforementioned methodology the appliances broke out of the palate and demonstrated a failure rate of 65%, probably because the appliances were placed in the incisors of the rats.

There have been concerns encountered in studies with palatal prostheses in rats related to weight loss and nutritional deficiencies, which affected the results of the studies evaluating virulence of microorganisms and biocompatibility. The effect of using palatal plates on the nutrition of the animals was evaluated by checking the weight of the animals at the time of the installation of the plate and comparing it with the weight after 14 days use of the plates. The wearing of the palatal plates fixed in the molars did not affect the animals' ability to eat a paste diet in this study, since no decline in the weight of the animals for any group was observed.

By means of morphologic criteria and HE technique, the results of the present study demonstrated that all the resilient materials tested *in vivo* in an animal usage test were well tolerated by tissues during the period evaluated. Considering the limitations of the biocompatibility tests, and the fact that the evaluation of morphologic criteria do not gives all parameters for toxicity, it may be necessary diverse group of tests before stating that a material is nontoxic [53]. For this purpose, future studies would be appropriate, as using imunohistochemical study of Ki-67 expression to the demonstration of the cell proliferation and evaluate inflammatory epithelial changes at molecular levels [61], [62].

## Conclusions

Within the limitations of this in vivo investigation and for the material evaluated in this study, the following conclusions can be drawn:

1. The soft reline materials tested did not cause significant histopathological changes in the rat palatal mucosa.

2. The post-polymerization heat treatment for Lucitone 550 was an effective method to reduce the histopathological changes in the rat palatal mucosa.
