# Correction: Histopathological Changes by the Use of Soft Reline Materials: A Rat Model Study

**DOI:** 10.1371/journal.pone.0106701

**Published:** 2014-08-20

**Authors:** The PLOS ONE Staff

FUNDUNESP is also one of the funders for this study. Please refer to the correct funding statement below:

This study was performed by MB as partial fulfillment of her PhD degree at the Ponta Grossa State University. This investigation was supported in part by Grants from the CAPES, Brazil (P.I. MB); Araucaria Foundation, Paraná, Brazil, 11386 (P.I. JHJ). In addition, this publication was supported by FUNDUNESP - Fundação para o Desenvolvimento da UNESP (0565/011/14-PROPe/CDC). The funders had no role in study design, data collection and analysis, decision to publish, or preparation of the manuscript.
